# Pinostilbene inhibits full-length and splice variant of androgen receptor in prostate cancer

**DOI:** 10.1038/s41598-023-43561-5

**Published:** 2023-10-04

**Authors:** Won Sik Shin, Seung Hyun Han, Kyung Won Jo, Yunje Cho, Kyong-Tai Kim

**Affiliations:** 1 Department of Life Sciences, Pohang University of Science and Technology (POSTECH), Pohang, 37673 Republic of Korea; 2 Hesed Bio Corporation, Pohang, 37563 Republic of Korea; 3 Generative Genomics Research Center, Global Green Research & Development Center, Handong Global University, Pohang, 37554 Republic of Korea

**Keywords:** Biochemistry, Cancer, Cell biology, Drug discovery

## Abstract

Prostate cancer is the most prevalent cancer in men worldwide and is promoted by the sex hormone androgen. Expression of androgen from the testis can be significantly reduced through castration. However, as most prostate cancer patients acquire castration resistance, additional therapeutic solutions are necessary. Although anti-androgens, such as enzalutamide, have been used to treat castration-resistant prostate cancer (CRPC), enzalutamide-resistant CRPC (Enz-resistant CRPC) has emerged. Therefore, development of novel treatments for Enz-resistant CRPC is urgent. In this study, we found a novel anti-androgen called pinostilbene through screening with a GAL4-transactivation assay. We confirmed that pinostilbene directly binds to androgen receptor (AR) and inhibits its activation and translocalization. Pinostilbene treatment also reduced the protein level and downstream gene expression of AR. Furthermore, pinostilbene reduced the protein level of AR variant 7 in the Enz-resistant prostate cancer cell line 22Rv1 and inhibited cell viability and proliferation. Our results suggest that pinostilbene has the potential to treat Enz-resistant CRPC.

## Introduction

Prostate cancer is the most common type of cancer found in men globally^1^. In 2015–2020, 109.9 per 100,000 men of all races and ethnic groups developed prostate cancer^2^. In the United States, the incidence of prostate cancer increased by 3% every year from 2014 to 2019^2^. Although prostate cancer has the second highest 5-year relative survival rate of 97%, due to the high incidence rate, the death toll per 100,000 people in 2015–2020 was 18.8, which is second highest after lung cancer^2^. In early stages, prostate cancer is subclinical and indolent^3^. However, it induces abnormal urination as a salient symptom^4^. Patients with this disease have prostatic hypertrophy that leads to frequent urination and nocturia^5^. In more advanced stages, patients suffer from retention of urine and pain in the back, hips, or limbs^6^.

Androgen receptor (AR) is a steroid hormone receptor activated by male sex hormones such as testosterone and dihydrotestosterone (DHT) and acts as a transcription factor by binding to the promoter in the nucleus^7^. Androgen receptor promotes the transcription of prostate-specific antigen (PSA) and transmembrane protease serine 2 (TMPRSS2) in the prostate^8,9^. PSA has been reported to promote the growth of AR-positive hormone-refractory prostate cancer cells such as CWR22rv1 and high passage LNCaP^10^. PSA promotes prostate cancer cell migration involved in the progression of prostate cancer^11^. Serum PSA level is an indicator of the disease^12^. It has been reported that TMPRSS2 plays an important role in prostate cancer cell invasion, tumor growth, and metastasis by activating matriptase and promoting extracellular matrix degradation^13,14^.

Because AR and male sex hormones contribute to prostate carcinogenesis, hormone therapy is an essential option for prostate cancer patients^15,16^. Orchiectomy, LHRH agonist, or antagonist treatment is often used as the first-line option to reduce the level of androgen produced in the testicles^17,18^. However, despite receipt of these treatments, most patients with metastatic prostate cancer eventually develop castration-resistant prostate cancer (CRPC) because of the small amounts of androgens produced in other organs such as the adrenal glands^19,20^. Anti-androgens such as enzalutamide, which inhibits AR level and downstream signaling by binding to AR ligand binding domain (LBD), were developed to treat CRPC^21,22^. Nevertheless, a significant number of CRPC patients is innately resistant (primary resistance) or acquire resistance (secondary resistance) to the anti-androgen treatment, hindering treatment of CRPC^19,23^. Several studies have pointed out that one of the causes of enzalutamide resistance is AR splice variants^24,25^. Most AR splice variants lack the C-terminal LBD and have an intact N-terminal transactivating domain and DNA-binding domain (DBD)^26^. AR variant 7 (ARv7, or AR3), which lacks LBD, is one of the major constitutively active splice variants that is not regulated by androgens or anti-androgens and can induce the expression of androgen-responsive genes^27,28^. It was reported that the mRNA expression of ARv7 is 20-fold higher in hormone-refractory prostate cancer than hormone-naïve prostate cancer^28^. For this reason, ARv7 is heavily involved in enzalutamide resistance (Enz-resistance) and hinders treatment of CRPC^25,29^. Therefore, it is necessary to develop drugs targeting Enz-resistant CRPC by inhibiting ARv7.

Pinostilbene, a natural product found in *Pinus sibirica*, is a monomethylated derivative of resveratrol that has anti-obesity, anti-inflammatory, hepatic protection, and neuroprotection capabilities^30,31^. Pinostilbene is also reported to inhibit proliferation of androgen-responsive human prostate cancer LNCaP cells, although its target and exact action mechanism are not known^32^.

In this study, we found that pinostilbene inhibits the transactivation of AR and investigated how it binds to AR. We explored how pinostilbene suppresses AR by analyzing its protein and mRNA levels. We found an inhibitory effect of pinostilbene on nuclear localization of AR and confirmed cytotoxicity and growth inhibition effects on prostate cancer cell lines. Furthermore, we revealed the efficacy of pinostilbene on 22Rv1, the Enz-resistant prostate cancer cell line. Here we present the effect of pinostilbene in inhibiting the action of ARv7, an AR variant.

### Pinostilbene binds to AR, reducing its activity

**Figure 1:**
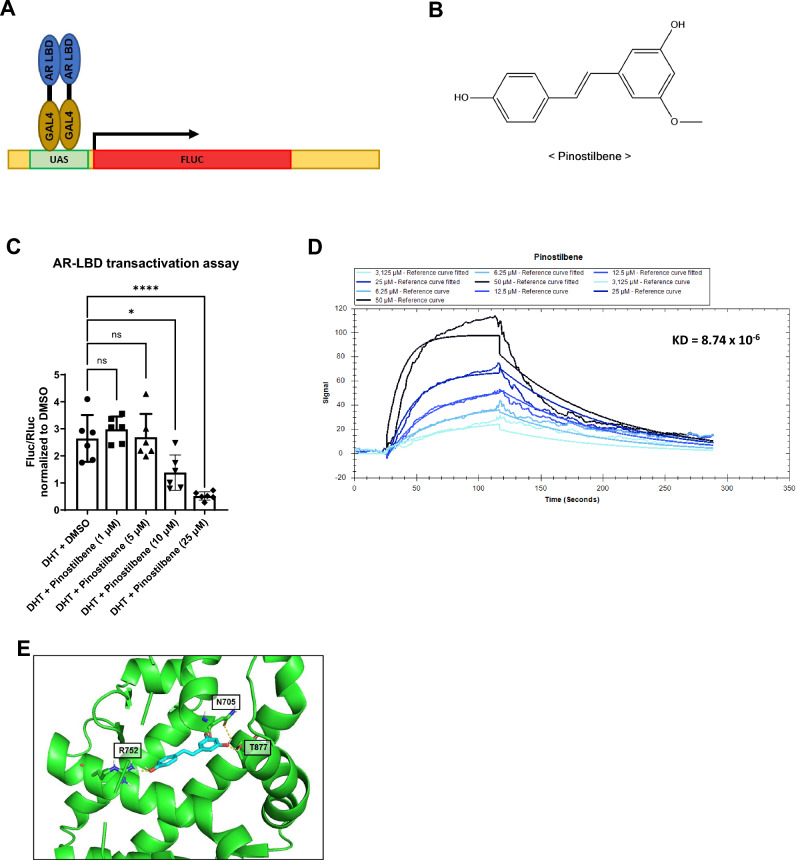
Interaction between androgen receptor and pinostilbene. (**A**) Schematic illustration of GAL4-AR LBD transactivation. (**B**) The chemical structure of pinostilbene. (**C**) UAS-Fluc, GAL4-hAR LBD (658–919), and Rluc plasmids were co-transfected in the HEK-293A cell line. Indicated concentrations of pinostilbene were applied with DHT for 12 h. Luciferase activity was measured in cell lysates. Fluc sactivities were measured and normalized to Rluc measurements (*n* = 6). (**D**) Direct interaction between GST-AR-LBD protein and pinostilbene was measured by SPR analysis. (**E**) In silico docking analysis. Pinostilbene (cyan) were docked with AR LBD (PDB ID: 2pip, green) by the Autodock vina in PyRx software^33,34^. Data represent the mean ± SD. *p < 0.05, ****p < 0.0001. Expression levels are relative to vehicle treatment (arbitrarily set to 1).

We performed an AR-LBD-GAL4 transactivation assay to identify novel compounds that can inhibit dimerization of AR by binding to its ligand binding domain (AR-LBD) (Fig. 1A). We co-transfected three plasmids (GAL4-hAR-LBD (658–919), UAS-Fluc, and Rluc) into the HEK-293A cell line. A neuroprotection compound library was treated with DHT to explore hit compounds that can reduce the luciferase activity induced by DHT.

Among the candidates, we found that pinostilbene effectively inhibits the luciferase activity of AR that is increased by DHT (Fig. 1B,C).

We tested the interaction of GST-AR LBD and pinostilbene through Surface Plasmon Resonance Analysis. Recombinant GST-AR LBD was successfully bound to the sensor chip for SPR analysis (Supplementary Fig. 1A). Pinostilbene showed binding activity with immobilized GST-AR-LBD in a concentration-dependent manner, as well as with enzalutamide (Fig. 1D, Supplementary Fig. 1B).

We predicted the binding sites of pinostilbene in AR LBD using the Autodock vina program in PyRx software^33,34^. Pinostilbene was predicted to interact with the AR-LBD amino acid residues Asn705, Arg752, and Thr877 (Fig. 1E), which have been reported as ligand-binding (testosterone-binding) residues^35^. These findings suggest that pinostilbene directly binds to AR-LBD and competes with ligands to inhibit transactivation activity of AR.

### Pinostilbene inhibits the expression of AR and downstream signals

**Figure 2:**
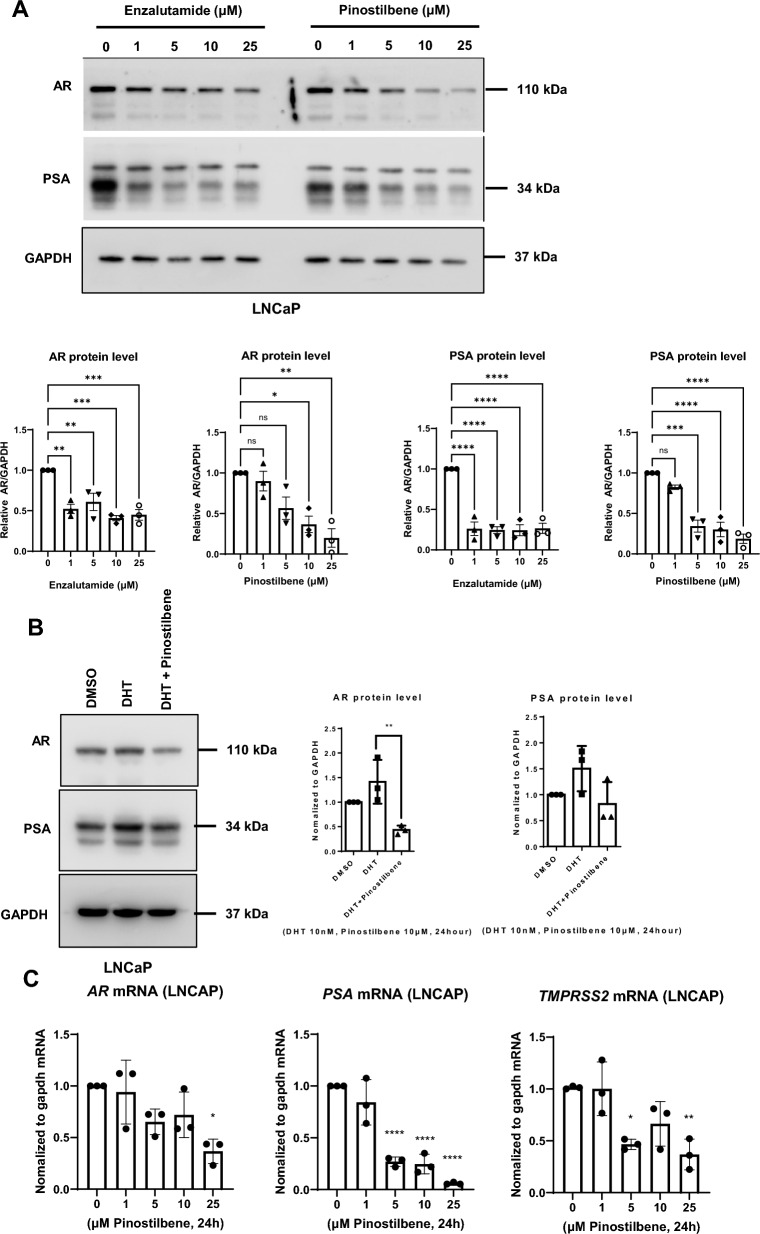
Effects of pinostilbene on AR expression in LNCaP. (**A**) Protein expression of AR, PSA, and GAPDH in LNCaP lysates after 24 h treatment of indicated concentrations of pinostilbene or enzalutamide (*n* = 3). (**B**) Antagonistic effects of pinostilbene against DHT. Protein expression of AR and PSA in LNCaP lysates after 24 h of 10 nM DHT treatment with or without 10 μM pinostilbene were analyzed by Western blot (*n* = 3). (**C**) Gene expression of AR, PSA, and TMPRSS2 in LNCaP cells after 24 h treatment with indicated concentration of pinostilbene was measured by RT-qPCR and normalized to that of GAPDH (*n* = 3). Data represent the mean ± SD. *p < 0.05, **p < 0.01, ***p < 0.001, ****p < 0.0001. Expression levels are relative to vehicle treatment (arbitrarily set to 1). Unprocessed original blots (low exposure) are presented in Supplementary Fig. 2.

As we confirmed that pinostilbene interacts with AR, we evaluated whether it affects AR and its downstream signals. We treated LNCaP, a human prostate cancer cell line that expresses AR, with enzalutamide or pinostilbene in a concentration-dependent manner. Western blot analysis showed that both enzalutamide and pinostilbene reduced protein expression of AR as well as PSA, which is a gene regulated by AR in LNCaP cells (Fig. 2A). When we treated DHT and pinostilbene together, pinostilbene decreased the protein level of the AR increased by DHT (Fig. 2B), indicating that pinostilbene antagonizes AR activation by DHT.

We performed RT-qPCR to confirm whether pinostilbene reduces the expression of downstream signals of AR. We found that both enzalutamide and pinostilbene effectively reduced mRNA expression of PSA and TMPRSS2 (Fig. 2C).

To determine how pinostilbene decreases the protein expression of AR, we treated proteasome inhibitor MG132 with pinostilbene to test the effects on protein stability of AR. AR level decreased by pinostilbene treatment was not rescued by MG132 treatment (Supplementary Fig. 3A), indicating that pinostilbene does not affect the ubiquitin–proteasome system-induced protein degradation of AR. On the other hand, pinostilbene as well as enzalutamide showed a tendency to reduce the mRNA expression of AR (Fig. 2C and Supplementary Fig. 2C). Taken together, these results indicate that pinostilbene inhibits transcription of AR and expression of its downstream genes.

### Pinostilbene inhibits nuclear localization of AR

**Figure 3:**
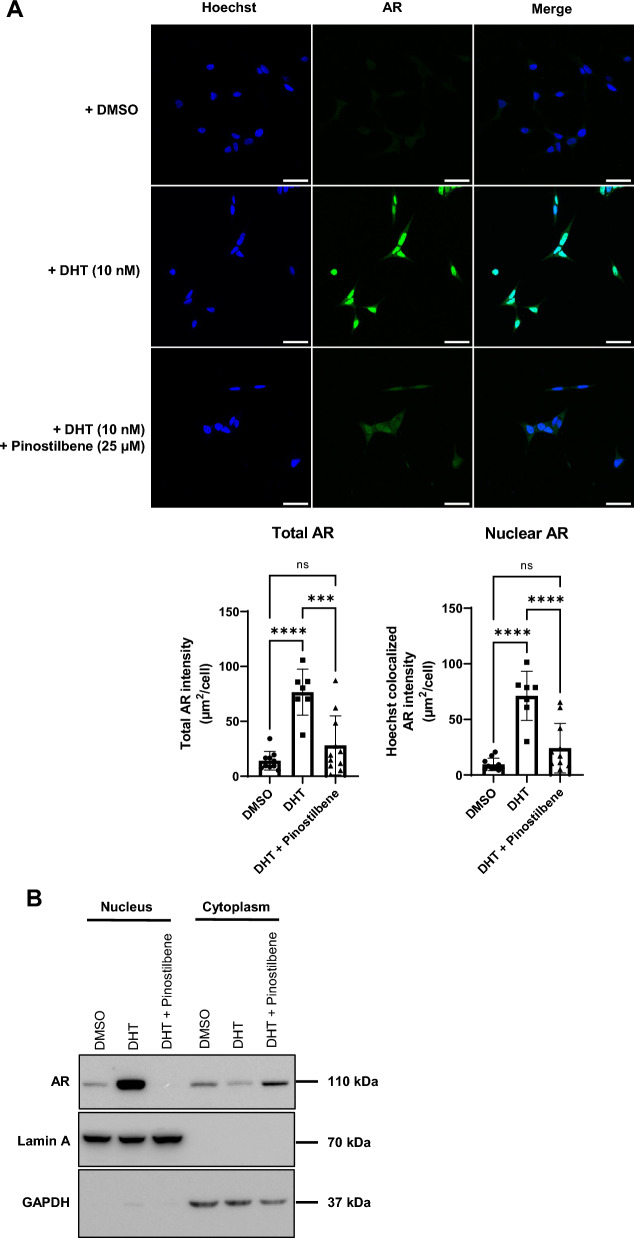
Effects of pinostilbene on AR localization. (**A**) (upper) Representative images of LNCaP cells under 12 h of serum starvation were treated with DMSO (*n* = 616 cells), DHT only (*n* = 317 cells) or DHT with pinostilbene (*n* = 233 cells) for 6 h. scale bar, 50 μm. (bottom) Quantification of total AR intensity and nuclear AR intensity. (**B**) Representative images of the protein expression of AR, GAPDH, and Lamin A in cytoplasm and nucleus of LNCaP cells treated with DHT only or DHT with pinostilbene (*n* = 3). Data represent the mean ± SD. ***p < 0.001, ****p < 0.0001. ns, not significant. Unprocessed original blots (multi-exposure) are presented in Supplementary Fig. 4.

When AR is activated by DHT, it forms a homodimer and is localized into the nucleus where it binds to androgen response elements (AREs) in the promoter DNA sequences to initiate transcription^36,37^. AR inhibitors such as enzalutamide or darolutamide antagonize AR and prevent its translocation into the nucleus by inhibiting AR binding of DHT^38,39^. We tested whether pinostilbene can inhibit nuclear localization by antagonizing AR. In the normal condition, AR was distributed evenly throughout the LNCaP cell (Fig. 3A). After treatment with DHT, LNCaP showed increased expression of AR mostly in the nucleus through colocalization of Hoechst staining. When DHT and pinostilbene were used together, the expression of nuclear AR was markedly reduced compared to the group treated only with DHT.

We also confirmed the suppression of nuclear localization of AR by pinostilbene through a cell fractionation assay. When LNCaP cells were treated with DHT, the protein expression of AR increased in the nuclear fraction. In contrast, when pinostilbene and DHT were used together, AR in the nuclear fraction was significantly decreased (Fig. 3B). Taken together, these data show that pinostilbene blocks the activation of AR by inhibiting its nuclear localization, resulting in suppression of AR gene expression.

### Pinostilbene inhibits the cell viability and proliferation of a prostate cancer cell line

The LNCaP cell line is a prostate cancer cell line whose growth is highly affected by the expression and activity of AR. Therefore, we tested whether pinostilbene can modulate the viability and growth of LNCaP through AR inhibition.

**Figure 4:**
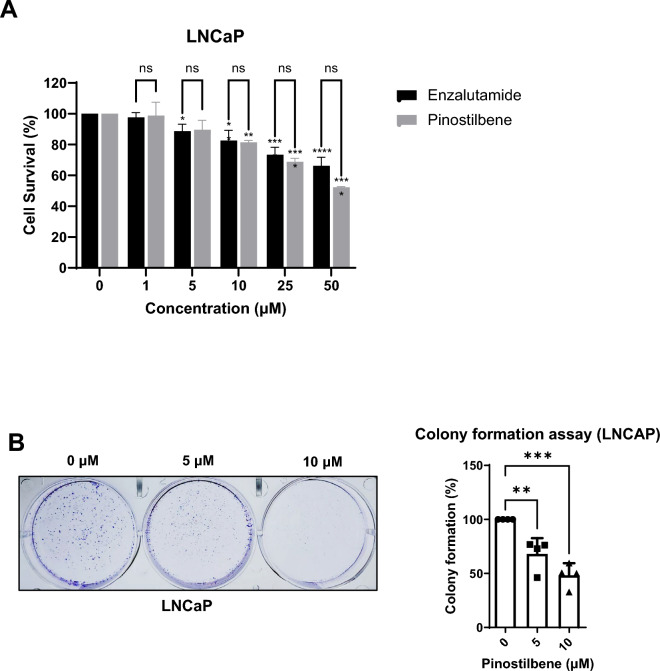
Cytotoxic and anti-proliferative effects of pinostilbene. (**A**) The viabilities of LNCaP cells were evaluated after treatment with indicated concentrations of pinostilbene and enzalutamide for 24 h. (**B**) Colonies of LNCaP formed after treatment with indicated concentrations of pinostilbene for 2 weeks were stained by crystal violet (CV). Stained colonies were destained by 20% acetic acid, and optical densities (ODs) of destained CV were measured at 595 nm. Data represent the mean ± SD. *p < 0.05, **p < 0.01, ***p < 0.001, ****p < 0.0001. ns, not significant. Expression levels are relative to vehicle treatment (arbitrarily set to 100%).

Pinostilbene showed significant cytotoxicity at 10 μM, similar to enzalutamide (Fig. 4A). However, in the HaCaT cell line, an immortalized human keratinocyte that does not express AR and that it is not a target cell for AR^40^, pinostilbene showed little cytotoxic effect (Supplementary Fig. 6A). To examine the cytotoxicity of pinostilbene in androgen-independent conditions, LNCaP cells were cultured in the media supplemented with charcoal-stripped serum in which various hormones including androgen are removed. Charcoal-stripped media condition reduced the protein level of AR in LNCaP (Supplementary Fig. 7B). LNCaP was less sensitive to pinostilbene in charcoal-stripped condition, which suggests reduction of AR desensitize the pinostilbene and pinostilbene has the androgen-dependent effect of pinostilbene (Supplementary Fig. 7C).

HaCaT cells formed colonies when treated with 10 μM pinostilbene, but colony formation of LNCaP cells was inhibited from 5 μM (Supplementary Fig. 6B, Fig. 4B). These findings suggest that pinostilbene has no significant effect on the growth of AR-independent non-cancer cells, while it has higher cytotoxicity in AR-dependent prostate cancer cell lines where it inhibits their growth, indicating that pinostilbene specifically affects the AR signaling pathway.

### Pinostilbene inhibits the Enz-resistant CRPC cell line by inhibiting AR variant 7

**Figure 5:**
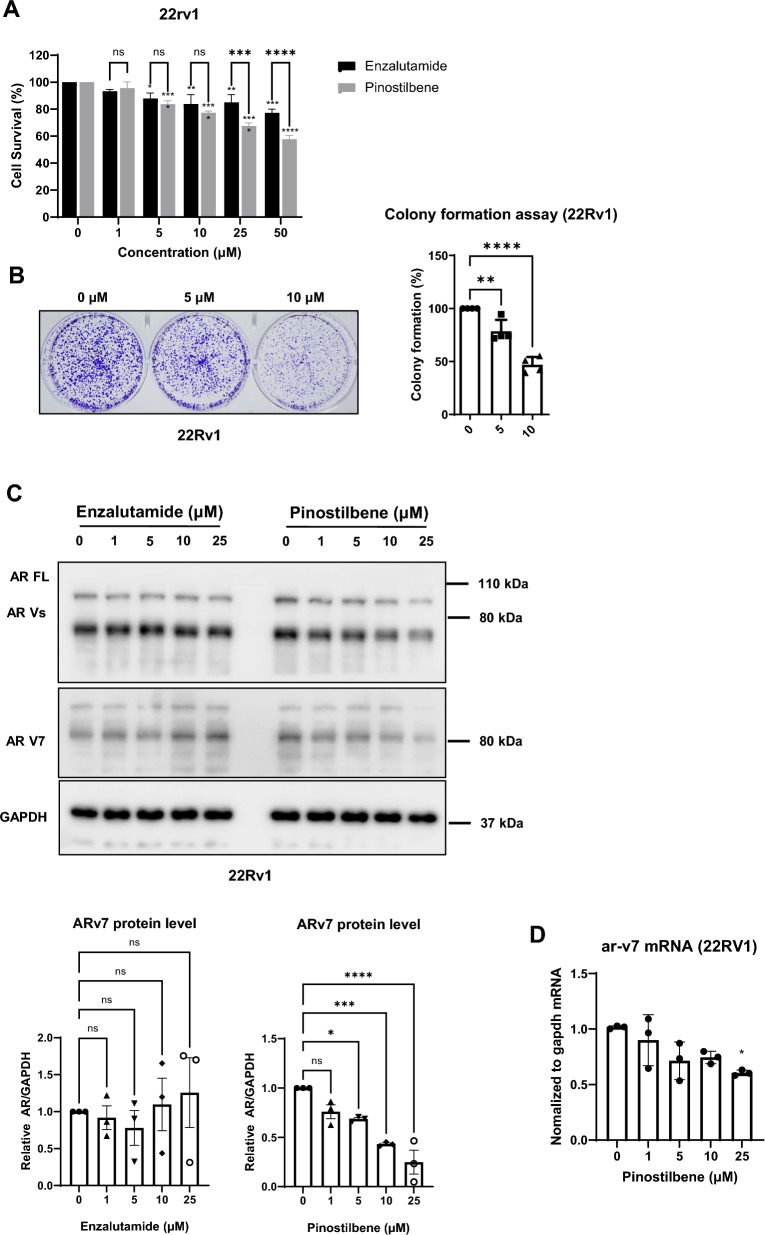
Effects of pinostilbene on ARv7 expression and 22Rv1 growth. (**A**) The viability of 22Rv1 cells was evaluated after treatment with indicated concentrations of pinostilbene and enzalutamide for 48 h. (**B**) Colonies of 22Rv1 formed after treatment with indicated concentrations of pinostilbene for 2 weeks were stained by CV. Stained colonies were destained by 20% acetic acid, and optical densities (ODs) of destained CV were measured at 595 nm. (**C**) Protein expression of AR, PSA, and GAPDH in 22Rv1 lysates after 24 h treatment with indicated concentrations of pinostilbene or enzalutamide (*n* = 3). (**D**) Gene expression of ARv7 in 22Rv1 cells after 24 h treatment with indicated concentrations of pinostilbene was measured by RT-qPCR and normalized to that of GAPDH. Data represent the mean ± SD. *p < 0.05, **p < 0.01, ***p < 0.001, ****p < 0.0001. ns, not significant. Expression levels are relative to vehicle treatment (arbitrarily set to 1 or 100%). Unprocessed original blots are presented in Supplementary Fig. 5.

We further tested whether pinostilbene inhibits the Enz-resistant CRPC cell line. Since ARv7 expression contributes to Enz-resistance of CRPC, we used 22Rv1, which is a well-studied Enz-resistant prostate cancer cell line that expresses both full-length AR and AR splice variants including ARv7^41,42^. Cell viability of 22Rv1 was analyzed through a CCK-8 assay. We found that pinostilbene has higher cytotoxicity than enzalutamide (Fig. 5A). The colony formation of 22Rv1 was significantly inhibited when treated with 10 μM of pinostilbene (Fig. 5B). These results suggest that pinostilbene inhibits the survival and proliferation of 22Rv1. When pinostilbene and enzalutamide were treated together in 22Rv1, combination effect could not be observed (Supplementary Fig. 7A). Charcoal-stripped condition completely diminished the cytotoxicity of enzalutamide in 22Rv1 cells. Although this condition rescued the viability of 22Rv1 treated with pinostilbene, it still retained concentration-dependent cytotoxicity, suggesting AR-independent cytotoxic effect of pinostilbene (Supplementary Fig. 7D).

We tested whether pinostilbene can also affect expression of the ARv7 protein. Enzalutamide did not affect the protein or mRNA level of ARv7 in 22Rv1 (Fig. 5C and Supplementary Fig. 2D). With pinostilbene treatment, the expression of ARv7 decreased in a concentration-dependent manner in the 22Rv1 cell (Fig. 5C). Pinostilbene also decreased the mRNA expression of ARv7 (Fig. 5D). However, as shown in the full-length AR, the failure of ARv7 to be rescued when treated with MG132 shows that pinostilbene does not affect the protein stability of AR (Supplementary Fig. 3B). This suggests that pinostilbene has additional inhibitory effects on AR variant-dependent tumors such as Enz-resistant CRPC.

## Discussion

Castration resistance has been an issue of interest in the field of prostate cancer for decades. The development of drugs that target androgens has been effective because adrenal androgens have the potential to promote prostatic cancer growth even if testicles are removed through orchiectomy^19,43^. Enzalutamide, a drug developed for treatment of CRPC, inhibits its activity by directly binding to the LBD of AR and competing with natural androgens such as testosterone and DHT^38,44^. However, emergence of Enz-resistance by AR variants has hindered the treatment of CRPC. In addition, enzalutamide showed various side effects and increased the risk for cardiac, infectious, metabolic, and respiratory disorders in the aged patient group (> 65 years)^45^. In phase III studies, the most frequent side effects of enzalutamide treatment were psychiatric and vascular disorders^45,46^. Therefore, it is essential to develop a new anti-androgen drug to counter the resistance to enzalutamide and reduce its side effects.

Decrease in the levels of full-length AR and ARv7 was not induced by proteasomal degradation because MG132 treatment did not rescue the reduction of AR protein by pinostilbene treatment (Supplementary Fig. 3). We initially found pinostilbene to be a candidate that binds to LBD of AR. However, pinostilbene can also reduce the protein and mRNA expression of ARv7, which does not contain LBD. Therefore, we predict that pinostilbene has multiple action mechanisms that affect not only full-length AR, but also ARv7. We are assuming that pinostilbene inhibits AR expression by suppressing the transcription of AR. Although the exact mechanism need to be elucidated, there is a report that showed pinostilbene treatment decreased the expression of key lipogenic transcription factor SREBP-1^47^. Moreover, SREBP-1 was reported to regulate AR promoter activity and transcriptional expression in prostate cancer cells^48^. Therefore, we hypothesize that pinostilbene may regulate the gene expression of AR by regulating SREBP-1. We are also aware of the possibility that pinostilbene may regulate alternative splicing of AR. Although molecular mechanism leading to alternative splicing of AR and how anti-androgens inhibit AR splicing have been unclear, recent studies have shown that it is mediated by various splicing factors such as KDM4B and hnRNP F^49,50^. In addition to these hypotheses, there is also a possibility that pinostilbene may regulate the mRNA stability of ARv7. In further study, we are going to test how pinostilbene affects the expression or activity of splicing factors or mRNA stability.

Confirmation of the antagonistic effect of pinostilbene against prostate cancer must be demonstrated through in vivo experiments. For development of novel treatment options for CRPCs, efficacy and stability of pinostilbene on Enz-resistant CRPC through xenograft mouse models using Enz-resistant cells such as 22Rv1 must be verified.

Resveratrol (3,4′,5-trans-trihydroxystilbene), a precursor to pinostilbene (3,4′-dihydroxy-5-methoxystilbene), has been shown to have anticancer activity against prostate cancer and to inhibit transcription activity of AR as well as its protein expression^51^. However, the usefulness of resveratrol in the body is limited by light, oxygen, and extreme pH conditions, which can cause trans–cis transformation or oxidation, reducing its bioavailability and bioactivity^52^. Chao et al. found that methylation of resveratrol's 5-hydroxyl group (to produce pinostilbene) increases its hydrophobicity and cell membrane permeability, resulting in a higher effective intracellular dosage, implying that pinostilbene is more effective in the body than resveratrol^31^.

Co-treatment with enzalutamide is also an important therapeutic option in CRPC. Studies have reported that the combination of enzalutamide and other anti-androgens that target ARv7 has potential in the treatment of Enz-resistant CRPC^53^. However, in cell level, we could not find any combination effect of enzalutamide and pinostilbene. Further validation of combination effects of pinostilbene and another anti-androgen are needed.

In this study, we found pinostilbene to be a novel AR antagonist through GAL4-AR-LBD transactivation assay. We confirmed that pinostilbene can directly bind to LBD of AR and can effectively modulate AR expression and expression of its downstream genes at the cellular level. We also found that treatment of prostate cells with pinostilbene inhibited nuclear localization of AR. Finally, we confirmed the AR-dependent cytotoxicity of pinostilbene in LNCaP cell lines. In addition, we confirmed that pinostilbene is cytotoxic even in the Enz-resistant prostate cancer cell line 22Rv1), and that it reduces expression of ARv7 and full-length AR. These results strongly suggest pinostilbene as an attractive option for treating Enz-resistant CRPC.

## Materials and methods

### Cell culture

LNCaP cells were cultured in RPMI-1640 (HyClone, Logan, UT, USA) supplemented with 10% fetal bovine serum (FBS; HyClone) and 1% penicillin–streptomycin (WELGENE, Republic of Korea) at 37 °C with 5% CO_2_. HEK-293A cells, HaCaT cells, and 22Rv1 cells were cultured in DMEM/High Glucose (Hyclone) supplemented with 10% FBS and 1% penicillin–streptomycin at 37 °C with 5% CO_2_.

### GAL4-AR-LBD transactivation assay

HEK-293A cells were transfected with GAL-hAR-658-919 (Addgene, Cat# 89082), UAS-promoter *Firefly* luciferase and *Renilla* luciferase reporter plasmids. After 24 h incubation, 100 nM 5α-Androstan-17β-ol-3-one (DHT, Sigma, Cat# A8380) were treated with or without different concentrations of neuroprotection library compounds (InterPham) or pinostilbene hydrate (Sigma, Cat# SML0098) for 12 h.

### Purification of recombinant protein

GST-tagged AR-LBD protein was expressed in *E. coli* BL21-Codon Plus (DE3) RIPL strain. Transformed *E. coli* were selected and grown in ampicillin agar plates at 37 °C. Selected colonies were picked and cultured in LB media until the OD 0.6–0.8. After ice incubation for 20 min, 0.5M IPTG was added for protein induction and incubated at 18 °C for 24 h. *E. coli* was resuspended with 1× cold PBS and disrupted by a sonicator. Cell lysates were centrifuged at 18,000 rpm, and the supernatants were obtained and incubated with glutathione-sepharose 4B agarose beads (GE Healthcare) at 4 °C for 24 h. After incubation, the lysates were centrifuged and supernatants were removed. Pellets were obtained and washed by 1× PBS with 1% Triton X-100. GST-AR-LBD proteins were eluted with GST elution buffer (50 mM Tris, 10 mM reduced glutathione, 120 mM NaCl, pH 8.0).

### Surface plasmon resonance analysis

SPR analysis was performed by IMSPR-Pro-HT (ICLUBIO). Brief procedure was performed by manufacture’s guide. Recombinant GST-AR-LBD proteins were immobilized on –COOH sensor chip (ICLUEBIO, Republic of Korea, Cat# HCCH101KX) through amine coupling using EDC (1-ethyl-3-(3-dymethylaminopropyl)carbodiimide)/NHS (N-hydroxysuccinimide) and remaining succinimide esters on sensor chip were blocked and de-activated by etahnolamine. Ligands in indicated concentration solved in 1% DMSO were flowed into the AR-LBD-immobilized chip. Then the chip was washed with 1% DMSO. Bound ligands were detached by 10 mM NaOH solution. The Response units (RU) was measured throughout all steps with iMSPR ProX (ICLUEBIO, Republic of Korea) using iMSPR software. Data evaluation and comparison, presentation were performed using TraceDrawer 1.9.2 (Uppsala, SWEDEN).

### In silico docking analysis

AR LBD (PDB: 2pip) structure file was downloaded from the Protein Data Bank (http://www.rcsb.org/). Already bound small molecules such as sulfate ions, ligand (DHT), and water molecules were removed. Then the macromolecule file was converted to PBDQT format for docking analysis. Grid square for ligand docking is set to cover all AR LBD. Molecular docking was performed by Autodock Vina module in PyRx software^33,34^. Visualization of docking pose and prediction of binding site between AR LBD and pinostilbene was performed by PyMOL software (Schrödinger, LLC.)

### Western blot

Cells were lysed by lysis buffer (20 mM Tris, 150 mM NaCl, 1 mM EDTA, 0.5% Triton-X 100, protease inhibitor (Roche)) and disrupted by a sonicator. Cell lysates were centrifuged at 15,000 rpm and soluble supernatants were separated. 15–30 μg of cell lysates were mixed with sample buffer (60 mM Tris, 25% glycerol, 2% SDS, 5% β-mercaptoethanol, 0.05% bromophenol blue) and separated by 10% SDS-PAGE. The proteins were transferred to nitrocellulose membrane. Membranes of each protein size were probed with the primary antibodies and the corresponding secondary antibodies. The signals were detected using LAS-500 (GE Healthcare) according to manufacturer’s protocol. All signals were quantified using Image J software^54^. Following antibodies were purchased: anti-AR (Cat# SC-7305, Santa Cruz Biotechnology), anti-AR-V7 specific (Cat# 68492S, Cell signaling), anti-PSA (Cat# ab53774, Abcam), anti-GAPDH (Cat# A300-641A, Bethyl), anti-Lamin A/C (Cat# 2032, Cell signaling).

### RT-qPCR

RNA was isolated using TRI reagent (Bio Science Technology). cDNA was synthesized using an ImProm-IITM Reverse Transcription System (Promega) and following the manufacturer’s instructions. Endogenous mRNA level was measured by A StepOnePlus Real-Time PCR System. The sequences of primers used in RT-qPCR are as follows:

- hAR, 5′-ATGGTGAGCAGAGTGCCCTA-3′ and 5′-GTGGTGCTGGAAGCCTCTCCT-3′
- hPSA, 5′-AAAAGCGTGATCTTGCTGGG-3′ and 5′-CATGACCTTCACAGCATCCG-3′
- hTMPRSS2, 5′-TCTAACTGGTGTGATGGCGT-3′ and 5′-GGATCCGCTGTCATCCACTA-3′
- hGAPDH, 5′-CCAAGGAGTAAGACCCCTGG-3′ and 5′-AGGGGAGATTCAGTGTGGTG-3′
- hARv7, 5′-CCATCTTGTCGTCTTCGGAAATGTTA-3′ and 5′-TTTGAATGAGGCAAGTCAGCCTTTCT-3′

### Cell fractionation

LNCaP cells were cultured with RPMI-1640 supplemented with 10% FBS and 1% penicillin–streptomycin at 37 °C with 5% CO_2_ for 24 h. Media was changed with FBS-free RPMI-1640 for 12 h. Serum-starved LNCaP cells treated with indicated chemicals for 6 h. Then cells were resuspended with lysis buffer (10 mM HEPES pH 7.4, 10 mM KCl, 0.05% NP-40, and protease inhibitors) and incubated for 20 min on ice. Samples were centrifuged at 14,000 rpm for 10 min at 4 °C. the supernatants were obtained as cytoplasmic fractions. Remaining pellets were washed with lysis buffer and centrifuged at 14,000 rpm for 10 min at 4 °C. Supernatants were removed, and pellets were obtained as nucleoplasmic fractions. Nucleoplasmic fractions were resuspended with lysis buffer and completely disrupted by a sonicator. Proteins of cytoplasmic and nucleoplasmic fractions were quantified in the same amount and subjected to Western blot.

### Immunocytochemistry

2 × 10^5^ LNCaP cells were cultured on 0.1% gelatin coated glass chip in 6 well plates. After treatments of indicated drugs, cells were fixed with 4% paraformaldehyde for 30 min and washed 3 times with 1× PBS. Cells were permeabilized with 0.5% NP-40 in PBS for 30 min and blocked with blocking solution (5% FBS, 2.5% BSA, 0.3% Triton X-100) for 2 h in room temperature. Mouse anti-AR antibody was added at 1:500 in blocking solution for 2 h, washed with PBS, and incubated with Goat anti-Mouse IgG (H + L) Cross-Adsorbed Secondary Antibody, Alexa Fluor™ 488 (Invitrogen, Cat# A11001) at 1:500 in blocking solutions for 2 h. Nuclei of cells were stained with 2 μg/ml of Hoechst 33342 for 30 min. Images were obtained using a laser scanning confocal microscope (FV3000; OLYMPUS). Image modification and quantification were performed using Image J software^54^.

### Cytotoxicity

1 × 10^4^ indicated cells were cultured in 100 μL media (RPMI-1640 or DMEM) with supplemented by 10% FBS or 10% charcoal-stripped FBS in 96 well plate. For cytotoxicity test with normal media condition, all cell lines cultured in the media supplemented with 10% FBS were cultured in 96 well for 24 h. For cytotoxicity test with charcoal-stripped condition, LNCaP cells are first cultured in the media supplemented with 10% FBS in 96 well. After 48 h, media were changed with 10% charcoal-stripped media and cultured for 72 h. 22Rv1 cells were cultured in the media supplemented with 10% charcoal stripped FBS for 1 week. Then 22Rv1 cells were cultured in 96 well plate with charcoal-stripped media. LNCaP cells were treated with indicated concentrations of reagents for 24 h. 22Rv1 or HaCaT cells were treated with indicated concentrations of reagents for 48 h. After incubation, cells were treated with 10 μl of CCK-8 solution and incubated 4 h at 37 °C. The absorbance was measured at 450 nm with INNO (LTEK, Republic of Korea) using INNO X software.

### Colony formation assay

1 × 10^4^ indicated cells were cultured in 6-well plate. After 24 h incubation at 37 °C with 5% CO_2_, cells were treated with indicated reagents for 2 weeks. Media were removed, and the cells were washed with 1× PBS. After the wash, the cells were stained with 0.5% crystal violet in PBS for 1 h with gentle shaking. Crystal violet solutions were removed, and the stained cells were washed with 1× PBS 3 times. Stained crystal violet dyes in cells were dissolved with 20% acetic acid and measured at 595 nm with Infinite 200 Pro NanoQuant using i-control 1.9 Magellan™ software (TECAN, Männedorf, Switzerland).

### Statistical analysis

All statistical analysis were performed using GraphPad Prism version 9.0. The unpaired Student’s t-test, one-way ANOVA, and repeated measure ANOVA were used to determine significance. A p-value p < 0.05, p < 0.01, p < 0.001 and p < 0.0001 are indicated by *, **, ***, and ****, respectively. All data are presented as mean ± SD or SEM.

### Supplementary Information

Supplementary Figures.

## Data Availability

The relevant data supporting the findings of this study are available within the article or from the corresponding author upon reasonable request.
