# Supplementary material for: Pinostilbene inhibits full-length and splice variant of androgen receptor in prostate cancer
Source: Sci Rep. 2023 Oct 4;13:16663. doi: 10.1038/s41598-023-43561-5 (PMC10550987; doi:10.1038/s41598-023-43561-5)
Supplement: Supplementary file 1 — Supplementary Figures. [file 41598_2023_43561_MOESM1_ESM.pdf]

Supplementary figure 1

A

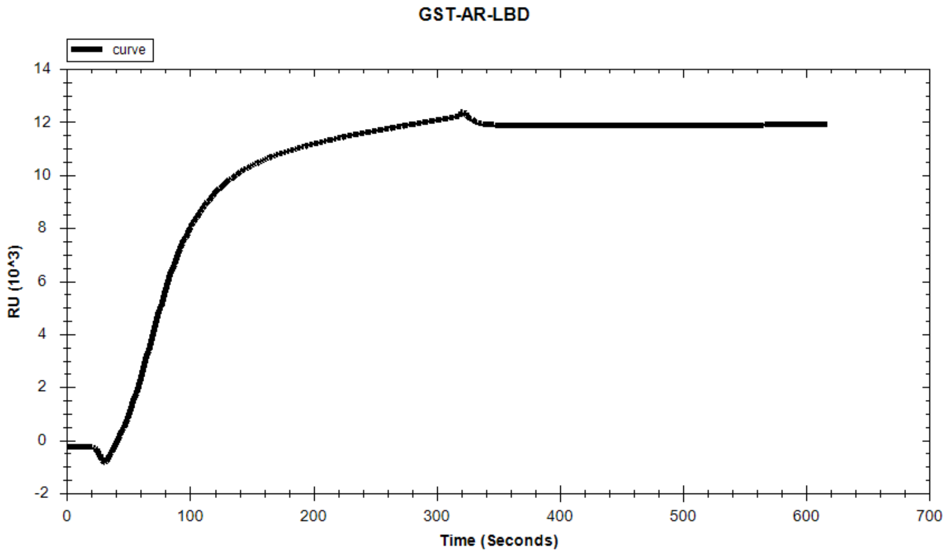

B

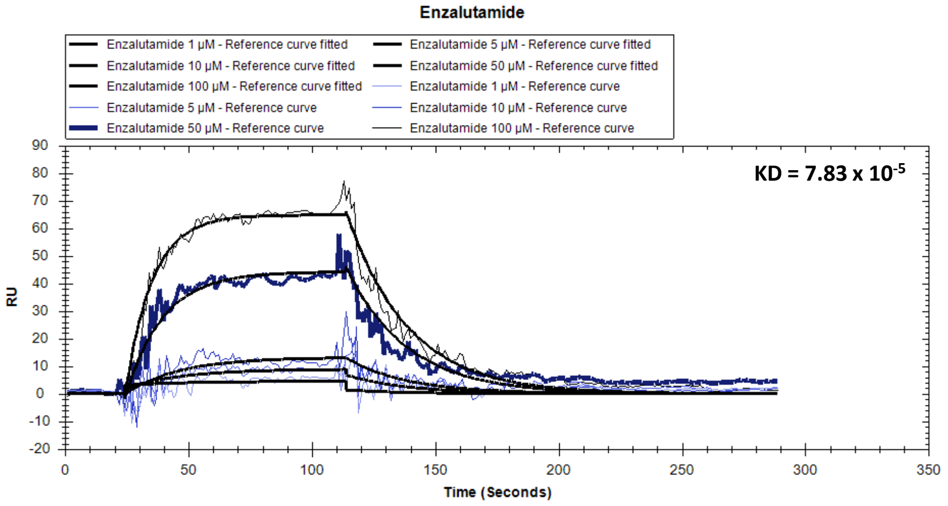

Supplementary Figure 1. Preparation and verification of surface plasmon resonance (SPR) system.

(A) Immobilization of GST-AR-LBD on a sensor chip.

(B) Direct interaction between GST-AR-LBD protein and enzalutamide was measured by SPR analysis.

Supplementary figure 2

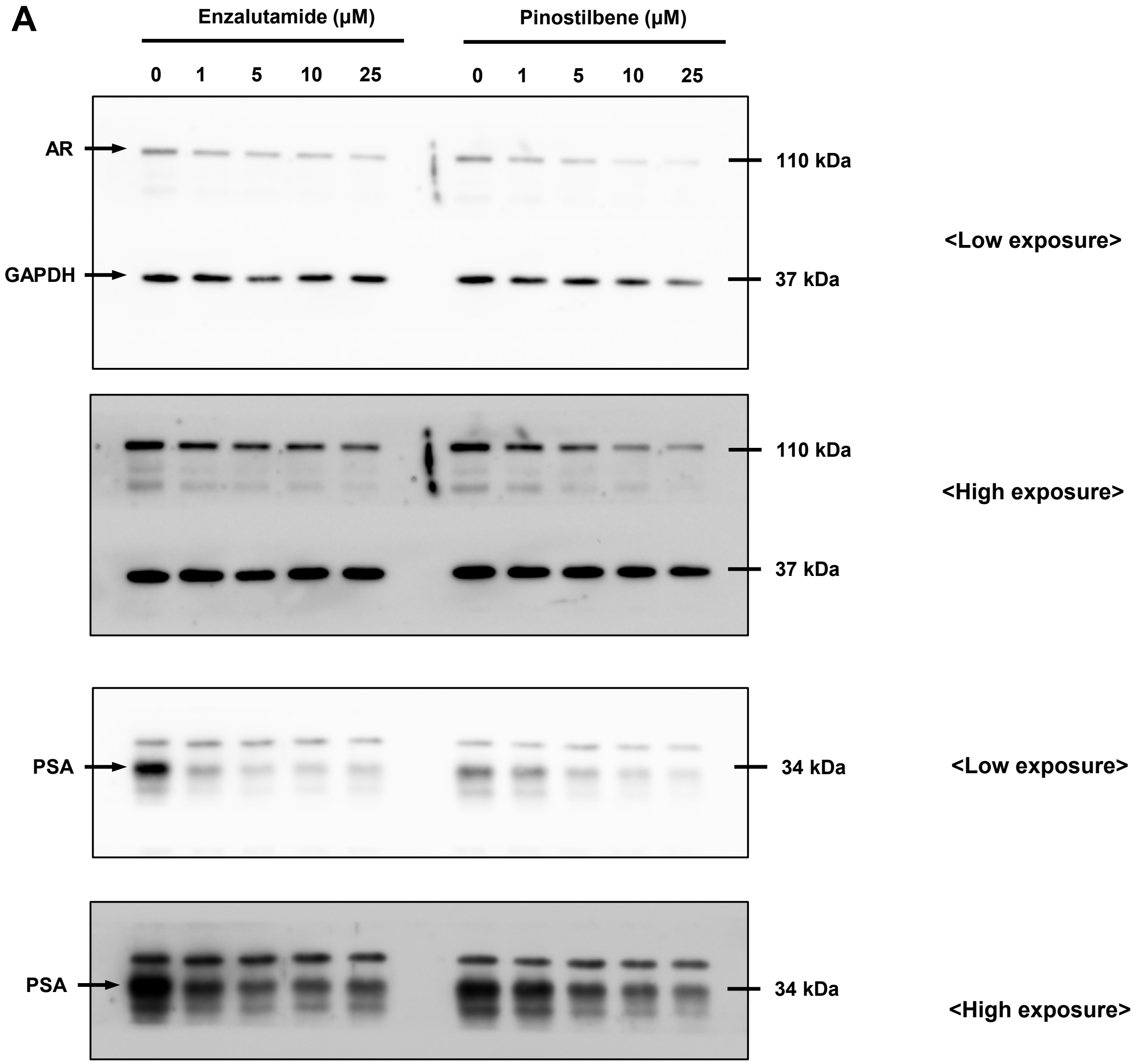

Supplementary Figure 2. Effects of pinostilbene on AR expression

(A) Representative images of Western blot images of main Figure 2A with multiple exposures. AR antibody, GAPDH antibody (up), and PSA antibody (down) were detected.

Supplementary figure 2

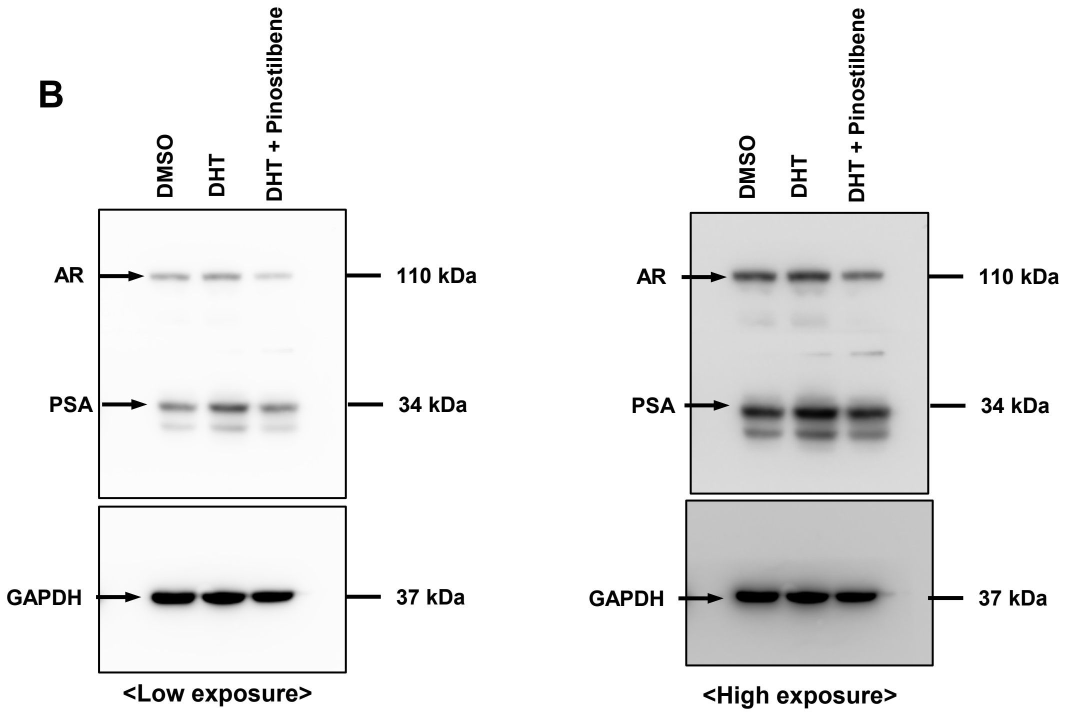

Supplementary Figure 2. Effects of pinostilbene on AR expression

(B) Representative images of Western blot images of main Figure 2B with multiple exposures. Total AR antibody and GAPDH antibody were applied first. After signals detected, blots were detached by stripping solution and applied by GAPDH antibody.

Supplementary figure 2

C

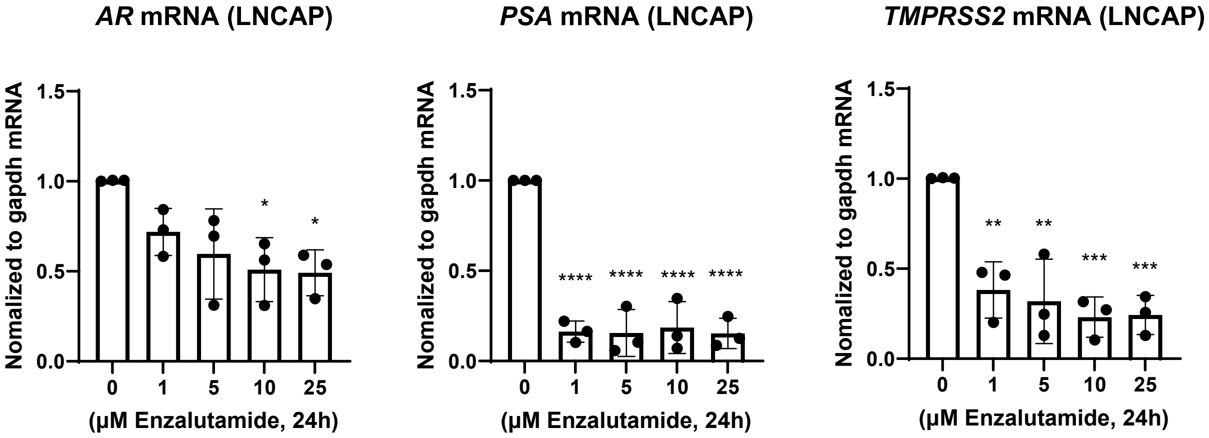

D

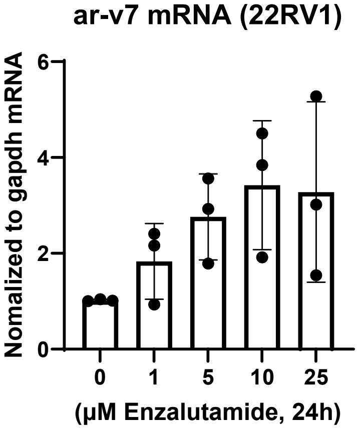

Supplementary Figure 2. Effects of pinostilbene on AR expression

(C) Gene expression of AR, PSA, and TMPRSS2 in LNCaP cells after 24 h treatment with indicated concentrations of enzalutamide was measured by RT-qPCR and normalized to that of GAPDH (n = 3). Data represent the mean ± SD. *p<0.05, **p<0.01, ***p<0.001, ****p<0.0001.

(D) Gene expression of ARv7 in 22Rv1 cells after 24h treatment with indicated concentrations of enzalutamide was measured by RT-qPCR and normalized to that of GAPDH. Data represent the mean ± SD.

Supplementary figure 3

A

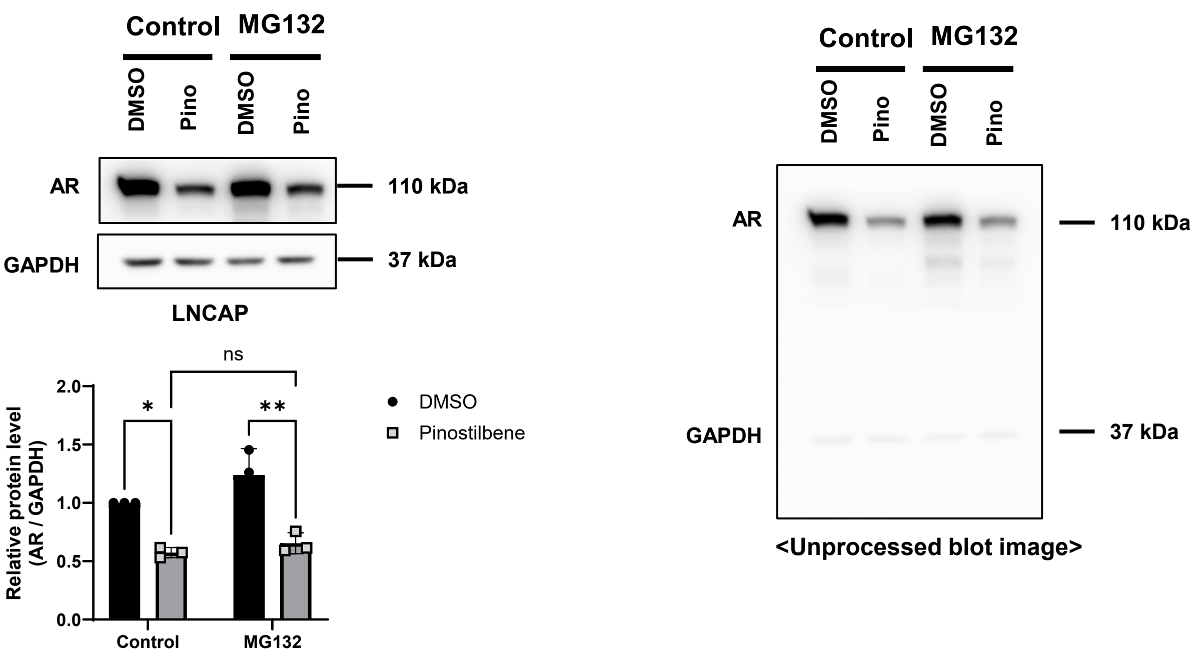

B

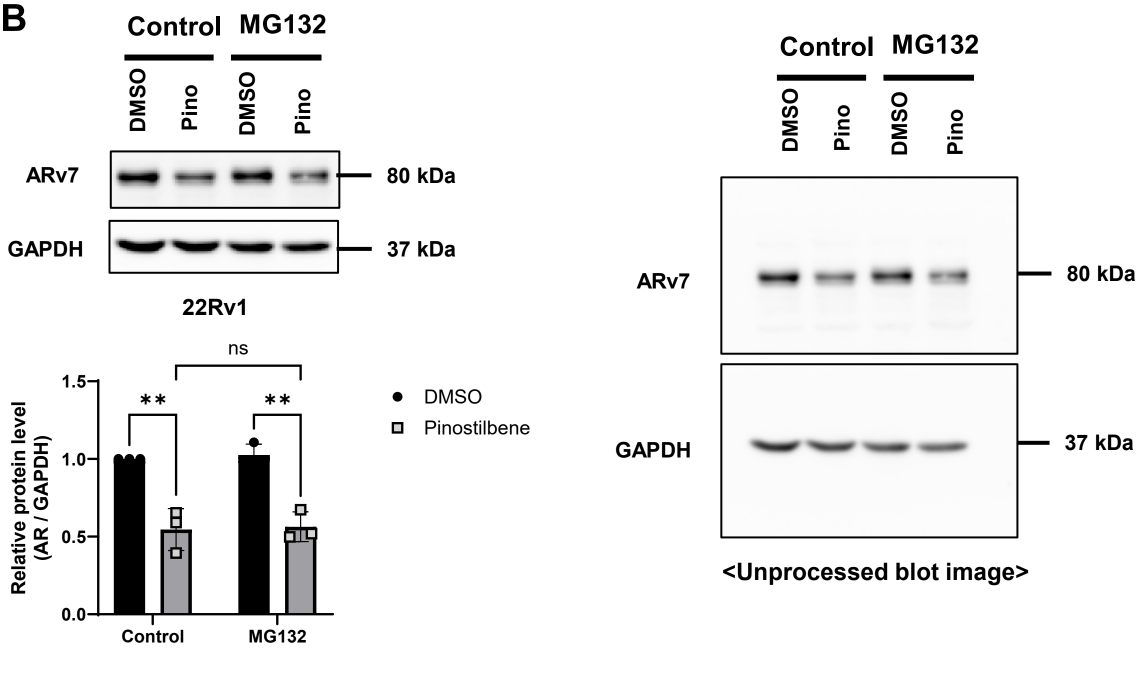

Supplementary Figure 3. Mechanism by which pinostilbene regulates androgen receptor.

(A-B) Protein expression of GAPDH and full-length AR or ARv7 in LNCaP **(A)** and 22Rv1 **(B)** after 6h co-treatment of MG132 with 25 μM pinostilbene after 18h treatment of 25 μM pinostilbene. Data represent the mean ± SD. *p<0.05, **p<0.01. ns, not significant. Expression levels are relative to vehicle treatment (arbitrarily set to 1). Unprocessed blots are shown on the right of each panels.

Supplementary figure 4

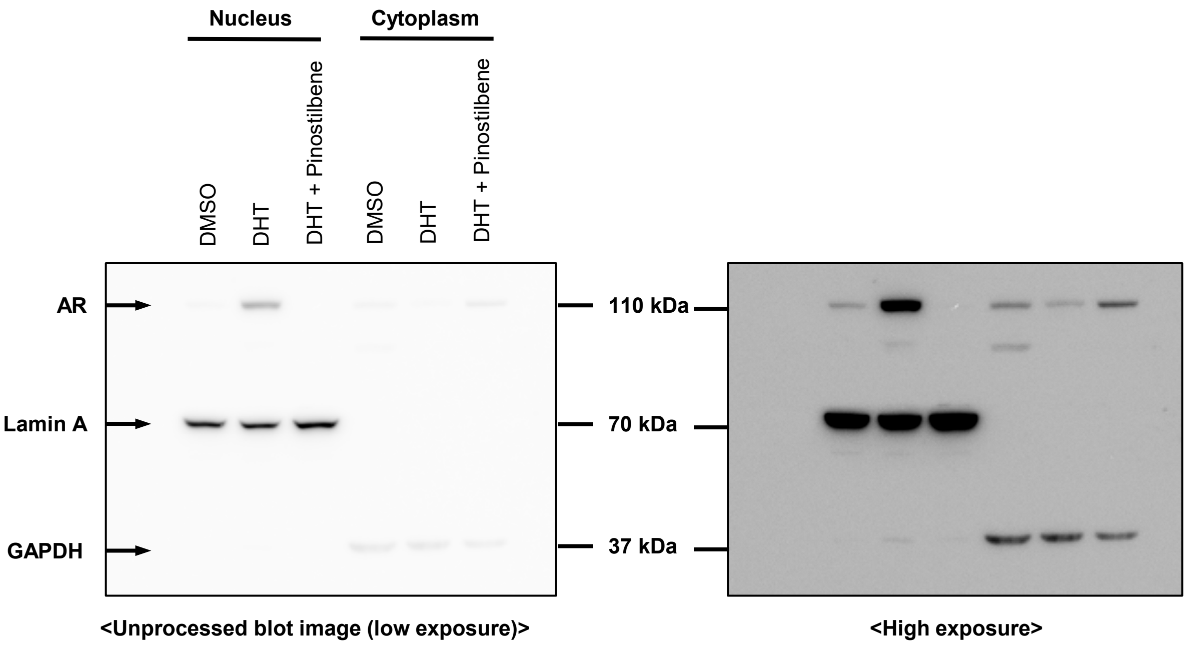

Supplementary Figure 4

Representative images of Western blot images of main Figure 3B with multiple exposures.

Supplementary figure 5

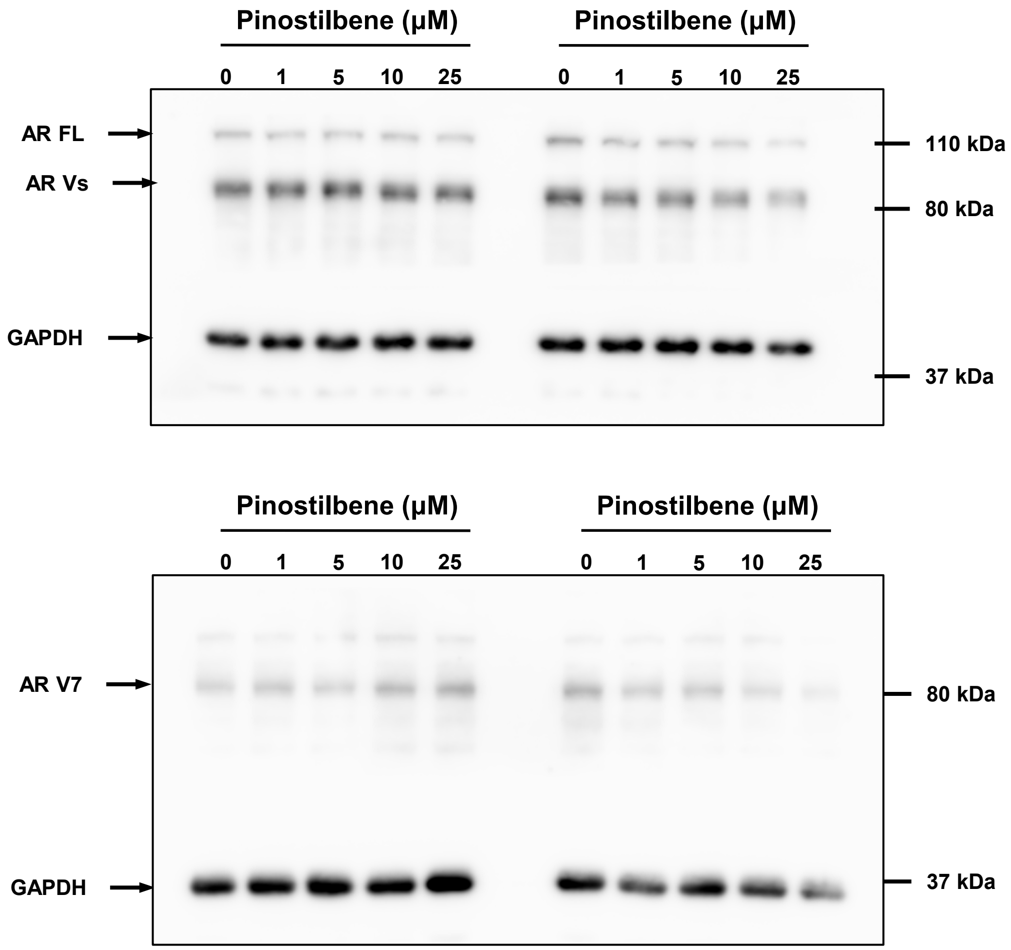

Supplementary Figure 5

Unprocessed original blot image (low exposure) of main Figure 5C.

Supplementary figure 6

A

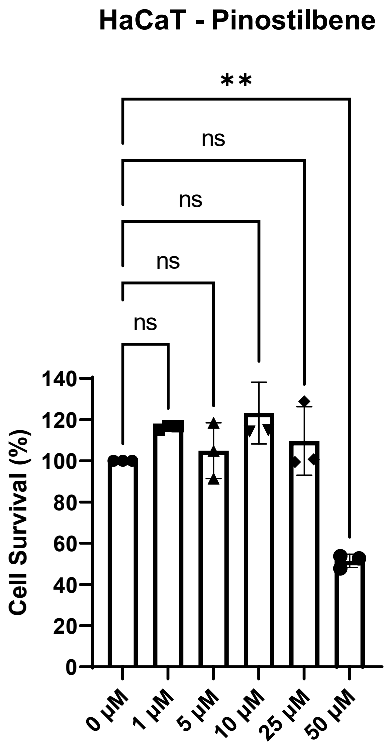

B

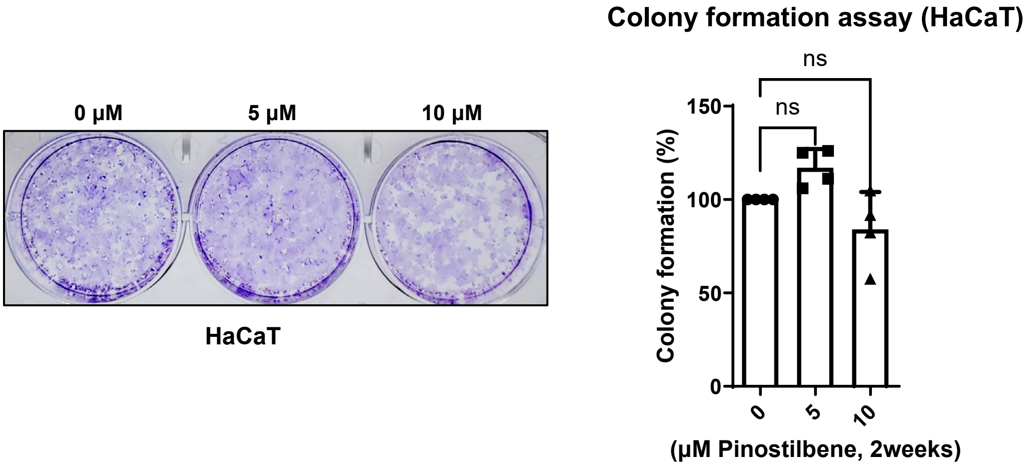

Supplementary Figure 6. Effects of pinostilbene on cell viability and proliferation of AR-independent HaCaT cell line.

(A) The viabilities of HaCaT cells were evaluated after treatment with indicated concentrations of pinostilbene and enzalutamide for 48 hours.

(B) Colonies of HaCaT formed after treatment with indicated concentrations of pinostilbene for 2 weeks were stained by crystal violet (CV). Stained colonies were destained by 20 % acetic acid, and optical densities (ODs) of destained CV were measured at 595 nm.

Data represent the mean ± SD. **$p<0.01$. Expression levels are relative to vehicle treatment (arbitrarily set to 100 %).

Supplementary figure 7

A

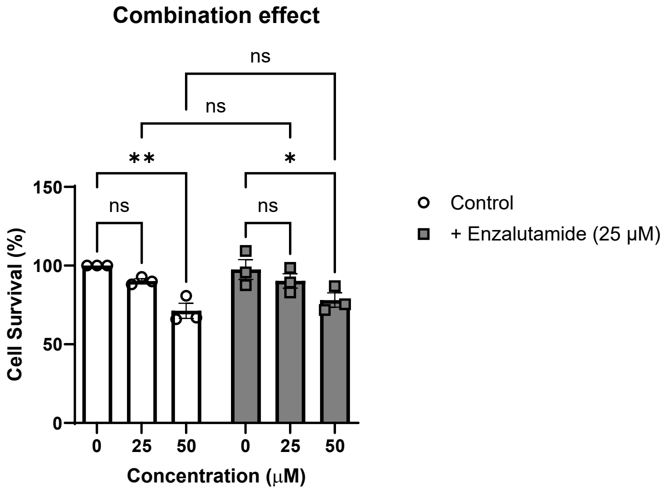

B

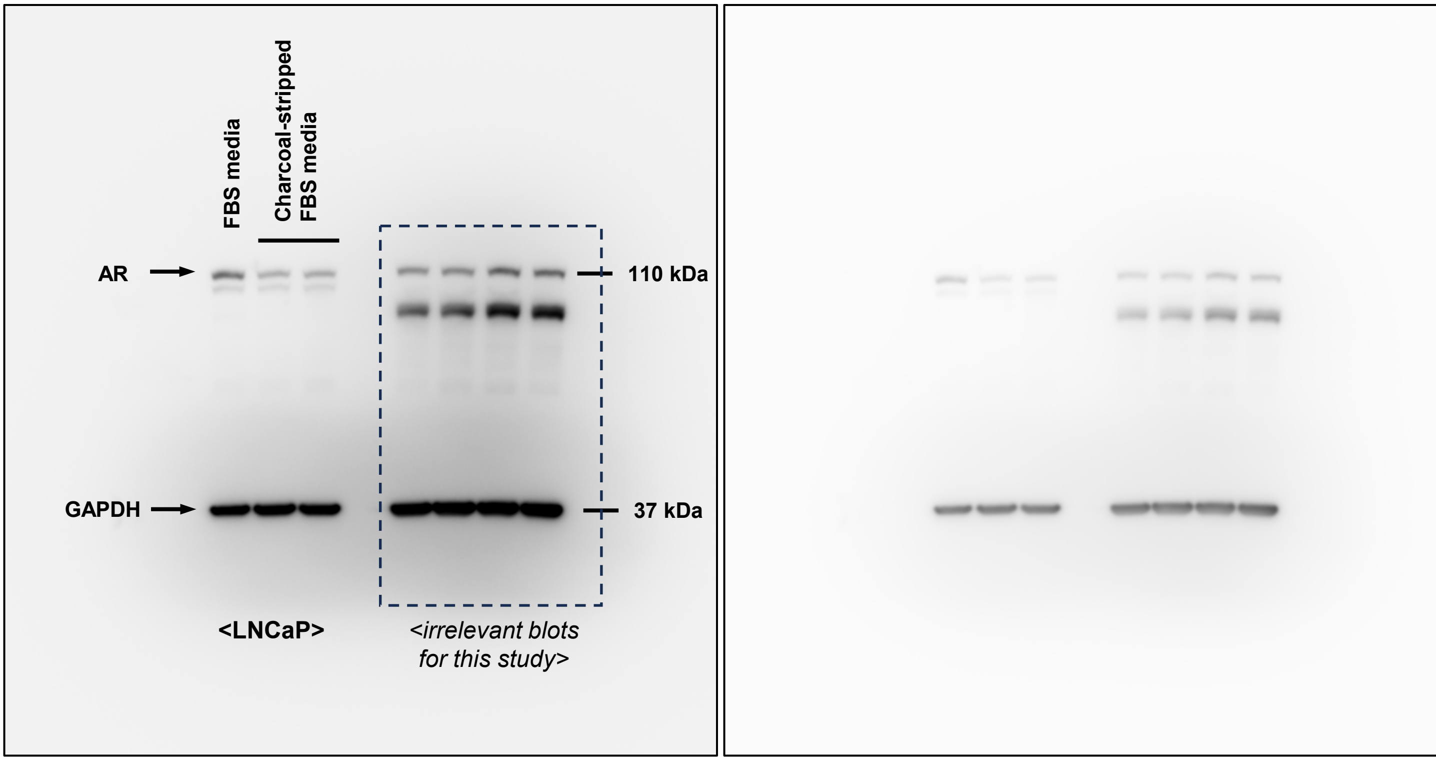

<High exposure> **<Unprocessed original blot image (low exposure)>**

Supplementary Figure 7. Effects of pinostilbene on cell viability and proliferation of prostate cancer cells.

(A) The viabilities of LNCaP cells were evaluated after treatment with indicated concentrations of pinostilbene and enzalutamide for 24 hours.

(B) (Left) Protein expression of AR and GAPDH in the lysate of LNCaP cultured in 10% FBS or 10% charcoal-stripped media for 24 hours. (Right) Unprocessed original blot image. Each blots were separately cropped by the size of each proteins (membrane edges of each blots are not shown even in high exposure image.)

Data represent the mean ± SEM. *p<0.05 and **p<0.01. Expression levels are relative to vehicle treatment (arbitrarily set to 100 %).

Supplementary figure 7

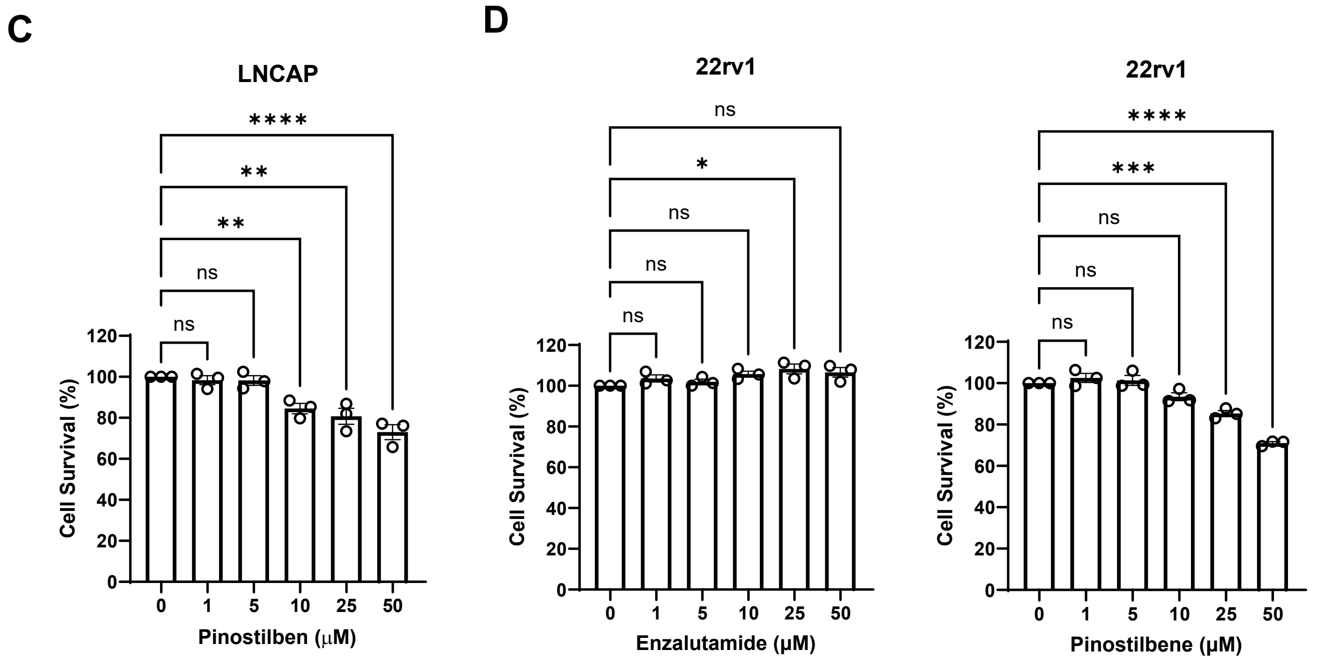

Supplementary Figure 7. Effects of pinostilbene on cell viability and proliferation of prostate cancer cells.

(C-D) The viabilities of LNCaP or 22Rv1 cells cultured in charcoal-stripped media were evaluated after treatment with indicated concentrations of pinostilbene for 24 or 48 hours respectively.

Data represent the mean ± SEM. *p<0.05, **p<0.01, ***p<0.001, and ****p<0.0001. Expression levels are relative to vehicle treatment (arbitrarily set to 100 %).
